# Recent approaches in whole cell pneumococcal vaccine development: a review study

**Published:** 2017-12

**Authors:** Mona Mohammadzadeh, Setareh Mamishi, Babak Pourakbari, Shima Mahmoudi

**Affiliations:** 1 Pediatric Infectious Diseases Research Center, Tehran University of Medical Sciences, Tehran, Iran; 2 Department of Pediatric Infectious Diseases, School of Medicine, Tehran University of Medical Sciences, Tehran, Iran

**Keywords:** *Streptococcus pneumoniae*, Vaccine, Protection

## Abstract

Despite the availability of relatively effective vaccines, *Streptococcus pneumoniae* still causes widespread morbidity and mortality. Current vaccines contain free polysaccharides or protein-polysaccharide conjugates, but do not induce protection against serotypes that are not included in the vaccines. Therefore, developing alternative vaccines is of high priority and importance. Several investigators have identified protective antigens common to pneumococci of many or all serotypes. Malley et al. in their study, have recommended unencapsulated whole cells, as an alternative vaccine, a number of such antigens unoccluded by capsule were presented in a native configuration in 2001. This review aimed at presenting this candidate of pneumococcal vaccine and results in an animal model.

## INTRODUCTION

*Streptococcus pneumoniae*, or pneumococcus, is an important human pathogen, and causes such diseases as sinusitis, otitis media, pneumonia, meningitis, and bacteremia (1). It has been estimated that 14.5 million episodes of severe pneumococcal disease occur each year worldwide, yielding 826,000 deaths of children aged 1 to 59 months (2). The polysaccharide capsule is the most important virulence factor of *S. pneumoniae*, and differences in the composition of the capsule are used as the basis to classify pneumococci into more than 90 serotypes (3–6). There are 2 types of licensed vaccines against invasive pneumococcal diseases, both based on the generation of antibodies against capsular polysaccharides (PS) (1). Anti-PS antibodies can opsonize the bacteria in a serotype-specific manner, leading to complement-dependent phagocytosis (7, 8). The first generation vaccine contains PS from 23 most prevalent serotypes and is indicated for the elderly (1). Because the response to PS is T-independent, this vaccine dose not induces memory, and due to the low immunogenicity of this vaccine, it is not recommended for children. Conflicting data have been reported on the efficacy of this vaccine and in fact, the use of the 23-valent PS vaccine has been questioned even for the elderly (1). The second generation vaccines are indicated for use in children and contain PS conjugated to carrier proteins, resulting in a T-dependent antibody response to PS (1). However, the widespread use of the second generation vaccines (PCV) has led to an increase in diseases caused by non-vaccine serotypes through a phenomenon known as serotype replacement (1). Serotype replacement in colonization has also been described (9, 10). These 2 vaccine categories have major disadvantages, which are as follow: (1) the free polysaccharide formulations fail to protect young children as the major risk group; and (2) the conjugate vaccines are based on a limited number of polysaccharides, are associated with serotype replacement, have restricted coverage, and are too expensive for use in the parts of the world with the greatest need, unless heavily subsided (11). Thus, developing alternative vaccines is still of high priority. These alternative vaccines included protein base vaccines, whole cell pneumococcal vaccines, and recombinant bacteria expressing pneumococcal antigens for the induction of serotype-independent protection against pneumococcal infection (1). The present work briefly summarizes the current knowledge on whole cell pneumococcal vaccines that are being investigated as potential vaccine candidates against pneumococcal infections.

### Advancements in serotype independent pneumococcal vaccines.

Malley et al. proposed developing a whole cell pneumococcal vaccine (WCV) as an economical means for presenting a multiplicity of common antigens in a native configuration without being masked by acapsule (12). This is a killed unencapsulated bacterium that was first tested as a nasal vaccine in mice, intending to reduce colonization by raising mucosal antibodies to many of the common antigens (13). In general, bacteria-like particles have an advantage in mucosal immunization (14, 15). One explanation is that particles co-presenting antigens and agonists of toll-like receptors are processed for immunity, whereas soluble antigens tend to be processed for tolerance (16). The absence of capsule allows the exposure of protein antigens on the pneumococcal surface to the immune system and thus, protection conferred by this vaccine can be sero-type-independent. In fact, nasal immunization with WCV, using cholera toxin (CT) or cholera toxin binding subunit (CTB) as adjuvant, was shown to confer protection against nasal colonization, with a variety of pneumococcal serotypes in mice (17).

### Whole cell vaccine preparation.

The whole cell pneumococcal vaccine is an inactivated cellular preparation of a non-encapsulated strain of *S. pneumoniae* derived from RX1; in which the *lytA* gene was deleted and the *ply* gene substituted for *pdT*; this formulation presents a combination of protective antigens common to all strains (12). Strain RX1 is a capsule-negative mutant derived from a pneumococcus capsular Serotype 2 (18). To grow to high concentrations, an autolysin (lytA)-negative mutant of RX1 (RX1AL) was used to prepare the killed vaccine preparations. This strain carries an erythromycin resistance gene (19). For vaccine production, RX1AL was grown at 37°C in Todd-Hewitt broth supplemented, with 0.5% yeast extract (THY) and 0.3 μg of erythromycin/mL to about 10^9^ cells/mL. The cells were washed and suspended in saline at 10% of the original volume. Samples were mixed 3:7 (volume/volume) with ethanol washed and resuspended in saline and frozen in small aliquots. When the killed vaccine preparation was cultured on blood agar, no viable bacteria were detected (lower limit of detection, 1 CFU/0.1 mL). The final vaccine mixture also contained CT, at 1 μg of CT per dose of vaccine (12).

### Mechanisms of protection.

To develop a more successful vaccine strategy, a better understanding of the mechanisms of immunity to pneumococcal colonization, which is the first step that leads to invasive disease is required (20). Several studies on the mechanisms of protection against nasal colonization of mice elicited by WCV have described the role of Th17 cells. Initially, it was proposed that this preparation would be administrated intranasally, with a strong mucosal adjuvant, inducing antibody-independent, CD4+ T cell-dependent immunity, and with production of IL-17, leading to the accelerated clearance of pneumococci from the nasopharynx (21). This protection against intranasal colonization was found to be effective against serotypes of pneumococci (17). More recently, it was found that systemic administration of the WCV preparation adsorbed to aluminum salts; and in addition to inducing IL-17 mediated protection against intranasal challenge, it induced antibody formation that protected mice in a model of lethal aspiration pneumonia, which is also independent of CD4+ T-cells (22). Protection was observed in mice that were deficient in producing the antibodies. Depletion of CD8+T-lymphocytes in vaccinated mice revealed no impact on protection, whereas depletion of CD4+T-lymphocytes abolished the effect of WCV on pneumococcal colonization (21). A strong negative correlation was obtained between the density of pneumococcal colonization in the nasopharynx of mice vaccinated with WCV and the levels of IL-17A in the blood; with non-detectable numbers of pneumococci in animals with high levels of this cytokine. In addition, protection was no longer observed in mice lacking the receptor for IL-17A (23). In an *in vitro* assay, IL-17A was found to enhance killing of pneumococci by neutrophils even in the absence of antibodies, which could be a mechanism for the protection conferred by WCV in mice with deficient in producing the antibodies. Using CT as an adjuvant, it was found that Th17 cells were the effectors in the protection against pneumococcal nasal colonization in mice immunized with the purified cell wall polysaccharide (CWPS). The zwitterionic charged motif of the pneumococcal CWPS provides a character to the polysaccharide that enables its presentation by B-cells, to T cells via MHC class II, resulting in the induction of IL-17A (24). The involvement of Th17 cells in protection against pneumococcal carriage stimulated the search for new vaccine formulations and antigens that could induce such response (1). More recently, the strategies pursued by Malley et al. to determine adequate WCV for human studies were reported (1). These include the use of an unencapsulated strain, which expresses a non-hemolytic derivative of pneumolysin and is autolysin-negative (25). Different methods for killing the bacteria were tested, with beta-propiolactone being the chosen agent (22, 25). In addition to the previous use of beta-propiolactone to produce human vaccines, this method allowed the retention of antigens in the killed bacteria, increasing the immunogenic potential of the preparations. Following a recommendation of the World Health Organization (WHO) (26), the vaccine was also tested through parental routes of immunization using aluminum hydroxide as an adjuvant. Among the important considerations that led to a move from nasal to parenteral immunization were the lack of safe adjuvants for nasal immunization and the homogeneity of the doses in infants eventually presenting copious nasal mucus (22). In addition, other mucosal routes, such as the oral or the sublingual methods, would require a considerably higher amount of antigen (25). The final protocol was found to confer protection against nasal colonization and lethal respiratory challenges in mice. The antibodies induced by the subcutaneous immunization of mice with WCV adsorbed to aluminum hydroxide showed cross-reactivity with different pneumococcal serotypes. The vaccine also induced Th17 responses against different pneumococcal isolates (27). The results supported the approval of a Phase 1 of the clinical trial for WCV, which is currently ongoing in the USA (1).

### Strains coverage.

Pneumococci are genetically and antigenically highly variable. Thus one should consider whether a vaccine made from a single strain could cover the extant variety of pneumococci worldwide and the variants that might arise with the pressure of WCV induced immunity. This variety cannot be dealt with animal protection studies, because relatively few pneumococcal serotypes reliably infect animals in models that recapitulate human diseases. Malley et al. have addressed this issue with 2 immunologic assays (1). The antibody reactivity was tested with the rabbit WCV antiserum, comparing it with preimmunization serum in a capture ELISA, which was conducted with a selection of 24 strains, including 15 serotypes and 12 multilocus sequence types (Of which, 14 were isolates from invasive diseases and 10 from carriage). WCV induced elevated titers against all the strains (The fold rise ranged from 10 to 140 times higher than the control serum) (2). Priming for Th17 response was tested by immunizing mice with WCV, isolating their splenocytes, and measuring IL-17A production by these cells in culture, when stimulated by various strains (27). Stimulation was consistent, indicating that the capsulation of the strains did not interfere with the expression of antigens able to stimulate T cells primed with the capsular vaccine cells. It is, thus, reasonable to expect broad coverage by WCA (13).

### Animal models and results.

Several studies have been conducted using the animal models on the WCV (Table 1). For the first time, Malley et al. have been examined WCV, applied intranasally with CT as an adjuvant in C57BL/6j mice (12). Immunization was delivered by gently restraining the unanesthetized mice and applying 10 μL to the nostrils a traumatically. Live pneumococcal preparations, (at a concentration of 10^6^ CFU/inoculation), killed Rx-1AL without or with CT, CT alone, or saline, was given 3 times at weekly intervals. One week following the last immunization, animals received 1 mg of rifampin in 0.25 mL subcutaneously. At 1 or 7 weeks later, the mice were challenged with 10^6^ CFU of *S. pneumoniae* type 6B applied as in the immunization. At 1 week after the challenge, the mice were euthanized by CO_2_ inhalation; the trachea was exposed and transected by careful dissection. An upper respiratory wash was done by instilling sterile, nonbacteriostatic saline retrograde through the transected trachea and collecting the first 6 drops (about 0.1 mL) from the nostrils. An animal was considered to be nasally colonized if ≥ 1 CFU/50 μL of washing fluid was detected on blood agar containing 2.5 μg of gentamicin/mL. In this study, 85% of the mice that received intranasal saline prior to challenge with live strain type 6B were colonized on Day 7. In contrast, mice that were immunized with killed Rx1AL plus CT were completely protected against nasopharyngeal colonization with *S. pneumoniae* type 6B. In the animals challenged with Rx1AL plus CT, 2 weeks after immunization, none was colonized, compared with saline controls. CT alone was partially protective, when animals were challenged 2 weeks post immunization, indicating that immunization with CT may provide nonspecific protection against colonization. When animals were challenged 8 weeks afterthe last immunization, the observed protection from CT was diminished, whereas the mice that received Rx1AL plus CT were still completely protected against colonization. The Rx1AL vaccine without CT was not significantly protective. Malley et al. have demonstrated that unencapsulated killed WCV administered intranasally can protect against nasopharyngeal colonization and invasive diseases.

**Table 1.** Summary of animal model studies on whole cell pneumococcal vaccine

**Author**	**Year**	**Animal model**	**Inoculation**	**Number of inoculation**	**Intervals of inoculation**	**Adjuvant**
Malley (13)	2001	C57BL/6J	Intranasal	3	1 week	CT
Malley (18)	2004	C57BL/6J	Intranasal	2	1 week	MALP-2 PLS MPLA CTB
Basset (34)	2007	C57BL/6J	Intranasal	3	1 week	CT
Bogaert (35)	2009	C57BL/6J	Intranasal	1	---	CT
Moffitt (28)	2012	C57BL/6J	Injection in the lower back	3	2	Al(OH)_3_
Moffitt (36)	2012	C57BL/6J	Intranasal	2	1 week	CT
Goncalves (37)	2014	BALB/C	Subcutaneous	2	2	Al(OH)_3_

While CT was an effective adjuvant in their model, due to toxicity, CT is not an acceptable adjuvant for humans. Therefore, to identify more appropriate adjuvants, in another study (17), they tested four candidates: mycoplasma-associated lipoprotein (MALP-2), a mucosal or systemic adjuvant in mice (28) that interacts with Toll-like receptor 2 (29); porcine lung surfactant (PLS) not containing surfactant protein A (30); *Bordetella pertussis* monophosphoryl lipid A (MPLA), resembling preparations studied in humans (31); and cholera toxin binding subunit (CTB), used mucosally in humans (32). For this purpose, WCV plus adjuvant were compared to adjuvant alone as well as to saline. Their study revealed that MALP-2, PLS and MPLA did not provide significant adjuvant activity. CTB gave only suggestive protective effects. CTB has been given safely by the intranasal route to humans and was effective as an adjuvant by this route (32).

Another study was conducted in 2007 by Basset et al. (33). They wished to test the hypothesis that protection derived from intranasal immunization with WCV is dependent on CD4+ T cells and independent of antibody. C57BL/6J mice were intranasally immunized 3 times at 1-week intervals with an adjuvant alone (CT) and WCV-CT. Four weeks after the third immunization, the mice were challenged intranasally with ∼10^6^ CFU of strain 0603. At 1 week after challenge, the mice were euthanized by CO_2_ inhalation; an upper respiratory wash was done by instilling sterile, nonbacteriostatic saline retrograde through the transected trachea and the first 6 drops were collected from the nostrils. An animal was considered colonized if at least 1 CFU/100 μL washing fluid was detected. To evaluate whether antibody-independent protection could also be elicited by WCV, they used C57BL/6J μMT/mice in which B-cell development was blocked at the pro-B stage. Immunization and challenge were delivered as described above. Immunization with WCV resulted in significant protection against nasopharyngeal (NP) colonization. Mice immunized with WCV had significantly lower density of pneumococcal colonization with strain 0603 than control mice that received CT alone. With respect μMT/mice, a significant reduction in NP colonization was observed in mice that received either the WCV compared to the mice that received the adjuvant alone, indicating that protection can be established in the absence of antibodies.

Since the target population of WCV is infants, it is important to determine whether this vaccine can confer protection following a single dose in infancy. So Bogaert et al. designed a study in which they used 6- to 8-day mice as infants, and 5- to 6-week-old mice as adults (34). For *in-vivo* comparison of the effects of vaccination on NP colonization, infant and adult mice were vaccinated just once. Mice were challenged intranasally with live strain 0603 and determined colonization 7 days after challenge. They found that colonization density was significantly lower in adult mice than in infant after challenge. They observed that, as in young humans, neonatal and infant mice are more susceptible to pneumococcal colonization than adult mice.

In another study which conducted in 2012, Moffitt et al. (27) examined the effect of WCV immunization against a panel of selected strains *in-vitro.* They evaluated the IgG titers in WCV immunization sera against live clinical isolates. C57BL/6J mice were injected 3 times at 2-week intervals. Each injection contained 100μg of WCV and 240 μg of Al(OH)_3_ as an adjuvant. The cross-strain immunogenicity of WCV was evaluated with a variety of strains. All the strains were tested by ELISA with live bacteria as capture antigen, determining the IgG antibody titer in sera of rabbits immunized with WCV, or co-housed rabbits injected with Al(OH)_3_ alone as controls. Higher titers were measured, against all strains tested in WCV-immunized serum over alum-control serum.

Identification of the protective components in the soluble fraction of the WCV has two important aspects. One of them is that the WCV consisting of over 2000 presumed expressed pneumococcal proteins. For production of WCV, knowledge of components of the vaccine would be very useful. Secondly, the success of the pneumococcal vaccines against invasive diseases can be ascribed to the remarkable impact of these vaccines on colonization. So identification and inclusion of antigens which are target to pneumococcal colonization in a vaccine would be advisable. In Moffitt et al. study (35) identification of immunogenic and protective proteins in the soluble fraction of an inactivated WCV were performed. Nearly, 4- to 6 week old C57BL/6J mice were immunized intranasally twice at 1-week intervals with WCV-CT and CT alone. Spleens were harvested from immunized animals 2- to 4 weeks after the last immunization for stimulations with WCV. Immunized animals that were subsequently challenged received an inoculum consisting of 10^7^ CFU of a clinical type 6B pneumococcal strain. These mice were euthanized by CO_2_ inhalation 1 week after the challenge; tracheal washes were obtained and assessed for density of pneumococcal colonization. To identify pneumococcal antigens, they used SDS-PAGE and Mass spectrometry. Subsequently, mice were intranasally immunized with the proteins. The Th17 responses following immunization with WCV were significantly higher compared to that of mice immunized with CT alone. Accordingly, animals immunized with the WCV were significantly protected from colonization. Among the antigens, SP2070 (Glucose-6-phosphate isomerase) was highly protective in their model and SP0862 (Ribosomal protein S1) and SP1534 (Putative manganese-dependent inorganic pyrophosphate) both significantly reduced colonization.

In Goncalves et al. study (36), In stituto Butantan, in collaboration with the Boston Group and with the support of PATH, developed the process for pilot scale production of this pneumococcal vaccine in 60-liter bioreactors under current Good Manufacturing Practices (cGMP). Groups of 10 BALB/C female mice were subcutaneously immunized with a sample obtained from drug substance batches (*S. pneumoniae* whole cell antigen-WCA), the Engineering Lot 005/09 Bulk, and the cGMP Lot009/10 Bulk, and its filled and formulated drug product (WCV), the cGMP Lot 1103054. These samples were diluted in phosphate buffered saline (PBS), using aluminum hydroxide as an adjuvant. Two injections with 2 weeks intervals were performed. To evaluate serum IgG, the animals were bled by retro-orbital puncture and challenged with live encapsulated *S. pneumoniae* 2 weeks after the last immunization. Significant IgG antibody titers against the WCA (Lot 005/09) were obtained with an apparent dose-response. The lower dose (1 μg) induced a non-significant survival of 30% after the challenge and the higher dose (10 μg) a survival of 85%. Potency of the cGMP Lot 009/10 Bulk was evaluated at a dose of 10 μg/animal at time zero *(T0)*, with samples used after freezing at −80°C, and after 12 months at −80°C. The serum antibody titers induced by the vaccine increased after 12 months of storage. The vaccine induced 80% of survival in immunized mice at time zero, and the protective potency was preserved after 12 months and maintained at −80°C, inducing 70% of survival; cGMP Lot 009/10 was formulated and filled to produce cGMP Lot 1103054, which was also evaluated for immunogenicity and potency under similar conditions, after 6 and 18 months at −80°C. In this case, a significant increase was observed in antibody titers against the whole cell vaccine after 18 months. The formulated and filled cGMP WCV product was protective, inducing 90% of survival after 6 months, and it still induced 100% protection in challenged mice after 18 months and stored at −80°C.

## CONCLUSION

Antigen-specific CD4+ T-cell responses might confer protection against pneumococcal colonization. Of course, multiple studies have to be done, and in particular IL-17A responses, can be detected in humans. Infant mouse macrophages show impaired innate and adaptive immunity to pneumococci, which might explain the increased susceptibility to pneumococcal colonization. The proteins identified through Moffitt study (35) can represent promising candidates for inclusion in a protein based vaccine. The non-encapsulated inactivated whole cell pneumococcal vaccine induces non-serotype specific immunity against several antigens common to all serotypes and can be manufactured by a simple procedure (12, 22, 25, 37). WCV elicited humoral and cellular immunogenicity to a comprehensive panel against varying serotypes of pneumococci. And finally, the whole cell pneumococcal vaccines produced under cGMP conditions were as immunogenic and protective as the same vaccine produced at bench scale. In light of the potential for omni-strain coverage from WCV immunization, as well as the economy of production, the plans for Phase I of clinical trials of the WCV are underway in the USA (27).
